# Transcriptome analysis reveals the molecular mechanism of γ-linolenic acid eradicating the biofilm of vancomycin-resistant *Enterococcus faecium*

**DOI:** 10.3389/fcimb.2025.1525581

**Published:** 2025-01-31

**Authors:** Ming Wei, Peng Wang, Tianmeng Li, Jun Liu, Yu Wang, Li Gu, Shuai Wang

**Affiliations:** Department of Infectious Diseases and Clinical Microbiology, Beijing Institute of Respiratory Medicine and Beijing Chao-Yang Hospital, Capital Medical University, Beijing, China

**Keywords:** vancomycin-resistant *Enterococcus faecium*, biofilm, γ-linolenic acid, eradication, molecular mechanism

## Abstract

**Introduction:**

Vancomycin-resistant *Enterococcus faecium* (VRE-fm) biofilms pose a significant clinical challenge due to the limited effectiveness of traditional antibiotics. This study investigates the potential of γ-linolenic acid (GLA) as a novel antibiofilm agent.

**Methods:**

Transcriptome analysis was performed on the V27 isolate, comparing cells in mature biofilms treated with and without GLA. The findings were further validated using qRT-PCR on six VRE-fm isolates and two *E. faecalis* isolates.

**Results:**

Transcriptome analysis revealed a significant downregulation in the expression levels of genes associated with biofilm formation, including *fruA*, *fruB*, *sgrA*, *lpxtg-cwa*, *tfpp*, *lafA*, *lafB*, *malP*, *fsrA*, and *fsrC’*, while a significant upregulation was observed in the expression of *fsrBD*. Validation by qRT-PCR in six VRE-fm isolates confirmed the significant changes in the expression levels of all genes except for *lpxtg-cwa*, with statistical significance. The expression of *bgsB* and *bgsA* genes, which are the homologs of *lafA* and *lafB* genes, along with the Fsr-regulated genes *gelE* and *sprE* in *E. faecalis*, were also found to be downregulated by GLA. In addition, KEGG analysis identified specific metabolic pathways that were significantly downregulated by GLA.

**Conclusion:**

GLA effectively targets multiple aspects of biofilm formation in VRE-fm, including the downregulation of key biofilm-related genes, the inhibition of quorum sensing systems, and the modulation of metabolic pathways. GLA emerges as a promising candidate for eradicating *Enterococcus* biofilms.

## 1 Introduction

Vancomycin-resistant *Enterococcus faecium* (VRE-fm) has become a major healthcare-associated pathogen, posing a significant threat due to its resistance to multiple antibiotics and the ability of biofilm formation (Wei et al., 2024). Extracellular polymeric substances (EPS) in biofilms provide structural integrity, protect them from environmental stresses, and facilitate communication between bacterial cells (Ch'ng et al., 2019). Biofilms promote *E. faecium* to form persisters and become viable but nonculturable (VBNC) (Lleò et al., 2005; Ayrapetyan et al., 2018). Biofilm formation not only increases the resistance of traditional antibiotics, but also is a critical enterococcal virulence factor, such as for infective endocarditis and urinary tract infection (Ch'ng et al., 2019). Therefore, alternative therapeutic approaches need to be developed against VRE-fm biofilm-associated infections.

Previous studies have shown that unsaturated fatty acids have antibacterial activity (Desbois, 2012; Yoon et al., 2018). However, due to the discovery and development of traditional antibiotics, research on the antibacterial properties of unsaturated fatty acids has been put on hold for a long time. With the increasingly serious problem of traditional antibiotic resistance, unsaturated fatty acids have been reconsidered as antimicrobial agents. Recent studies have shown that unsaturated fatty acids can also inhibit or eradicate biofilms formed by various microbial pathogens (Inoue et al., 2008; Kim et al., 2018; Ramanathan et al., 2018; Cui et al., 2019; Wang et al., 2022; Khan et al., 2023). Therefore, unsaturated fatty acids are very promising next-generation antibacterial agents for the treatment and prevention of biofilm-related infections.

Our previous research has found that essential fatty acids (EFAs) not only inhibit the growth of VRE-fm, but also inhibit its formation of biofilms, and even eradicate its already formed biofilms (Wei et al., 2023). In terms of eradicating biofilms, we found that γ-linolenic acid (GLA) can reduce the expression of the *atlA* gene, which is responsible for facilitating the release of eDNA (Xie et al., 2022; Wei et al., 2023). However, inhibition of eDNA release alone may not be enough to achieve a biofilm eradication rate of more than 60%, so we believe that there are other mechanisms that need to be further studied.

This study aims to unravel the molecular mechanisms by which GLA eradicates biofilms formed by VRE-fm using RNA sequencing (RNA-seq) technology. By providing a comprehensive snapshot of the transcriptome, RNA-seq can be used to identify genes that are differentially expressed during biofilm eradication by GLA, as well as to provide insights into the regulatory pathways involved (Willett et al., 2019; Tatta et al., 2023). In our preliminary studies of six clinical isolates of VRE-fm, GLA showed the highest biofilm eradication efficacy against isolate V27. Therefore, we selected VRE-fm isolate V27 for subsequent transcriptomic analysis. We hypothesize that GLA exerts its antibiofilm effect by modulating the expression of genes involved in various stages of biofilm formation, including adhesion, EPS production, and cell signaling. Understanding these mechanisms is crucial for developing novel strategies to combat biofilm-associated infections caused by VRE-fm.

## 2 Materials and methods

### 2.1 Strains and culture conditions

A total of six clinical VRE-fm isolates (V05, V06, V08, V09, V22, and V27) with moderate biofilm formation ability (Wei et al., 2023), and two *E. faecalis* isolates (efa105 and efa106) with strong biofilm formation ability were collected from the Department of Infectious Diseases and Clinical Microbiology, Beijing Chao-Yang Hospital, Capital Medical University (Beijing, China). The isolates were identified using matrix-assisted laser desorption/ionization-time of flight (MALDI-TOF) (VITEK-MS; bioMeírieux, France; IVD version 3.0). These isolates were refreshed from frozen stocks at -20°C and inoculated twice on Columbia blood agar at 35°C for 24 h before all experiments.

### 2.2 RNA preparation for sequencing and qRT-PCR assay

The mature biofilms of the six VRE-fm isolates (V05, V06, V08, V09, V22, and V27) were established according to the previous study (Wei et al., 2023). After the biofilms were treated with or without GLA for 24 h, the *E. faecium* cells were collected by centrifugation (5000 × g for 10 min). Total RNA from the *E. faecium* cells was extracted using the RNA extraction reagent kit (Tiangen Biotech, Beijing, China) according to the manufacturer′s instructions. The quantity and quality of the isolated RNA samples were determined using a NanoDrop One Spectrophotometer (Thermo Scientific, Waltham, MA, USA). The extracted RNA samples of V27 isolate underwent quality control and library preparation for transcriptome sequencing.

### 2.3 Clustering and sequencing of the V27 treated with and without GLA samples

TruSeq PE Cluster Kit v3-cBot-HS (Illumia) was used for performing the clustering of the index-coded samples on a cBot Cluster Generation System according to the manufacturer’s instructions. After cluster generation, an Illumina Novaseq platform was used for sequencing the library preparations and 150 bp paired-end reads were generated.

### 2.4 Data quality control of the V27 treated with and without GLA samples

Raw data of fastq format were processed through in-house perl scripts. Then, clean data were obtained by removing reads containing adapter, low-quality reads, and reads containing N base from raw data. At this step, Q20, Q30, and GC content of the clean data were calculated. All the subsequent analyses were based on high-quality clean data.

### 2.5 Reads mapping to the reference genome

*E. faecium* 1,231,408 (GenBank: GCA_000157615.1) was selected as a reference strain in this study. Its genome files were downloaded as the reference genome from the website directly. The Bowtie2-2.2.3 software was used for building an index of the reference genome and aligning clean reads to the reference genome.

### 2.6 Quantification of the gene expression level of the V27 treated with and without GLA samples

HTSeq v0.6.1 software was used for counting the reads numbers mapped to each gene. And then Fragments Per Kilobase of exon model per Million mapped fragments (FPKM) of each gene was calculated based on the length of the gene and reads count mapped to this gene. FPKM is currently the most common method for estimating gene expression levels.

### 2.7 Differential expression analysis of the V27 treated with and without GLA samples

The read counts were adjusted by the edgeR program package through one scaling normalized factor for each sequenced library before differential gene expression analysis. DEGSeq R package (1.20.0) was used for differential expression analysis of two conditions. Genes with an edgeR *P* value < 0.05 and |log2(Fold change)| ≥ 1 were set as the threshold for significantly differential expression.

### 2.8 GO and KEGG enrichment analysis of differentially expressed genes of the V27 treated with and without GLA samples

Gene Ontology (GO) enrichment analysis of differential expression genes was implemented by the GOseq R package, which was used for correcting the gene length bias. GO IDs with *P* value < 0.05 were considered significantly enriched by differential expression genes.

KEGG (Kyoto Encyclopedia of Genes and Genomes) is a database resource for understanding high-level functions and utilities of the biological system from molecular-level information, especially large-scale molecular datasets which are generated by high-throughput experimental technologies (http://www.genome.jp/kegg/). The KOBAS software was used for testing the statistical enrichment of differential expression genes of the V27 isolate in KEGG pathways.

### 2.9 Differential expression genes validation by qRT-PCR assay of the 6 VRE-fm isolates

RNA (~2 μg) from each sample was reverse transcribed for the synthesis of cDNA using SuperScript™ III First-Strand Synthesis SuperMix (Invitrogen, Carlsbad, CA, USA). The PowerUp SYBR Green Master Mix (Applied Biosystems, Life Technologies, Austin, USA) was used for quantitative reverse transcription PCR (qRT-PCR) assay in an Applied Biosystems 7500 real-time PCR system (Applied Biosystems, Waltham, MA, USA). The amplification reactions were carried out in a 20 µL volume including 10 μL of 2× PowerUp SYBR Green Master Mix, 1 μL of forward primer, 1 μL of reverse primer, 6 μL of DNase/RNase-Free water (Tiangen Biotech, Beijing, China), and 2 μL of the synthesized cDNA. The reaction conditions were 95°C for 3 min with 1 cycle, then 40 cycles (95°C for 15 s, and 60°C for 1 min) for DNA template amplification. Gene expression was normalized with the reference gene *gdhA* using 2^−ΔΔCT^ method (Livak and Schmittgen, 2001). The forward and reverse primers used for the detection are shown in **Supplementary Table S1**.

Statistical analysis for qRT-PCR assay was performed using the IBM SPSS Statistics 25.0 software program (IBM, Armonk, NY, USA). Data were expressed as means ± standard deviation (SD) of three independent experiments. Student’s t-test was used for statistical comparison between the two groups. The standard F-test was used to test whether two populations had the same variance. A *P* value < 0.05 was considered statistically significant.

### 2.10 Evaluation of the efficacy of GLA in eradicating the biofilm of *E. faecalis* isolates

Biofilm eradication was assessed using two clinical isolates of *E. faecalis*. Mature biofilms were pre-formed in trypticase soy broth supplemented with 2% glucose (TSBG) for 24 hours. After incubation, non-adherent cells were removed by washing twice with PBS. Subsequently, TSBG containing 1 mM GLA was added to the wells. A control group was treated with fresh TSBG medium. Microtiter plates were incubated at 35°C for an additional 24 hours, and biofilm mass was quantified using the crystal violet staining method. The biofilm eradication rate (%) was calculated using the following formula: Eradication (%) = (OD_control_ - OD_sample_)/OD_control_ × 100% (Wang et al., 2022).

### 2.11 Biofilm-related gene expression in *E. faecalis* isolates analyzed by qRT-PCR

RNA extraction, cDNA synthesis, amplification reaction, and statistical analysis for the two *E. faecalis* isolates treated with and without GLA were performed using the same method as described above. The primers of efa-*gdhA*, *bgsA*, *bgsB*, efa-*malP*, *gelE*, and *sprE* genes are shown in **Supplementary Table S2**.

## 3 Results

### 3.1 RNA-Seq quality control and analysis

The Illumina sequencing generated a total of 15,199,804 and 15,939,512 reads for the V27 treated with GLA and V27 treated without GLA (control) samples, respectively. After eliminating low-quality reads, reads containing adapter and N base, 14,793,234 and 15,581,624 clean reads were obtained. The PHRED quality scores of Q20 of the filtered reads were 95.30% and 97.69% for the V27 treated with GLA and V27 treated without GLA samples, respectively, which confirmed the presence of high-quality sequencing reads in the transcriptome dataset. The reference genome of *E. faecium* 1,231,408 (GenBank: GCA_000157615.1) was selected for reference-based assembly of the transcriptome. The RNA-Seq analysis was performed by mapping filtered reads for each sample to the reference genome. The data denoted that 71.97% and 87.77% of the reads were successfully mapped to the reference genome, while 64.01% and 77.47% of reads were uniquely mapped for the V27 treated with GLA and V27 treated without GLA samples, respectively. Post read mapping the transcript quantification was performed to obtain the expression details of the transcripts for each sample.

The number of transcripts with FPKM values in the ranges of 0-1, 1-3, 3-15, 15-60, and >60 was 637 (20.15%) and 529 (16.74%), 143 (4.52%) and 47 (1.49%), 414 (13.10%) and 442 (13.98%), 481 (15.22%) and 641 (20.28%), and 1486 (47.01%) and 1502 (47.52%) for the V27 treated with GLA and V27 treated without GLA samples, respectively. The Venn diagram showed that 2519 (95.53%) genes were co-expressed (**Supplementary Figure S1**), and the Pearson correlation was 0.879 between the two samples.

### 3.2 Differential expression genes

**Figure 1:**
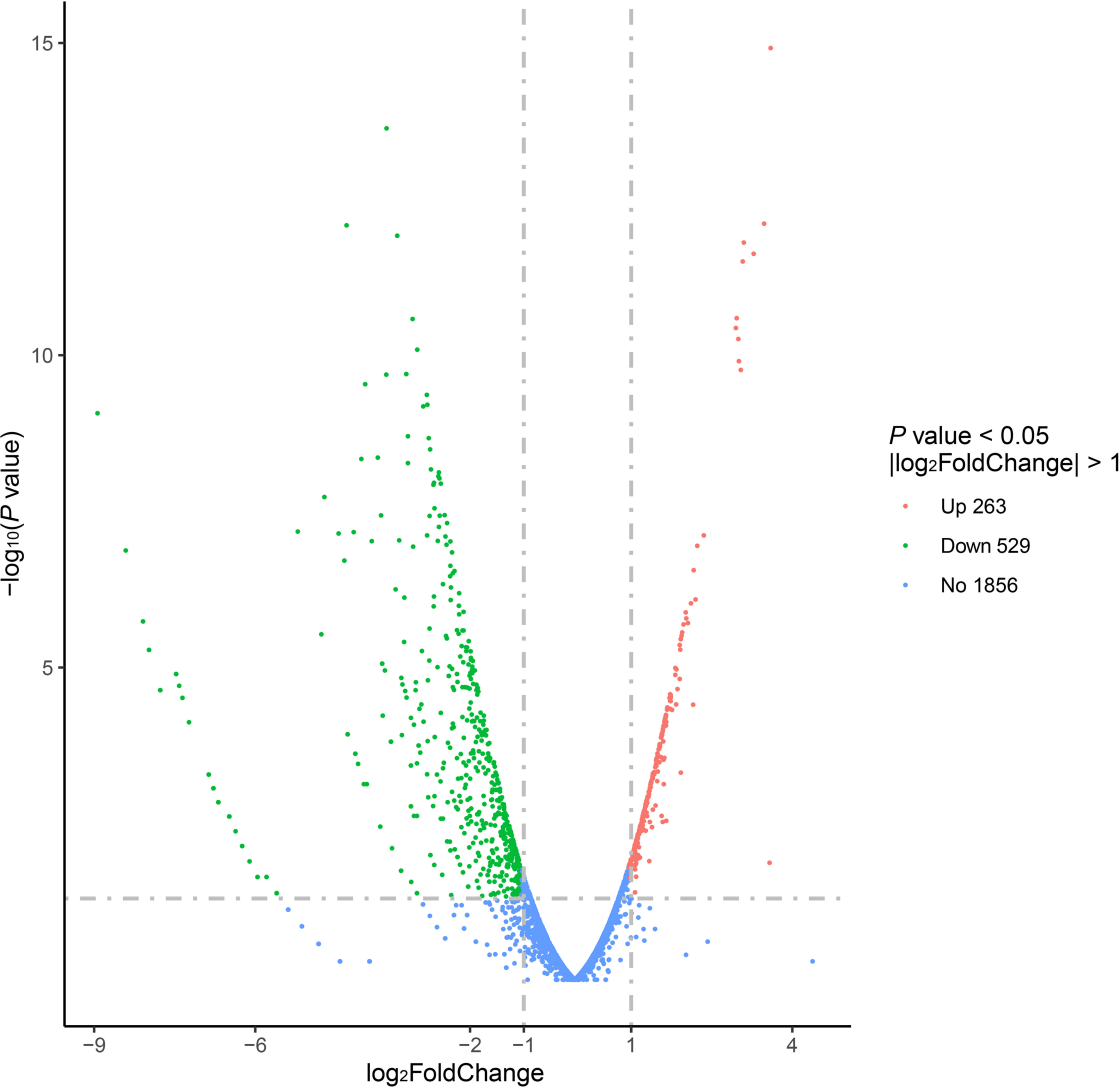
The volcano plots of differential expression genes for the V27 treated with GLA *vs.* V27 treated without GLA.

Comparison of the transcriptome of the V27 treated with GLA to the V27 treated without GLA samples revealed that a total of 792 genes were being differentially expressed [|log2(fold change)| ≥ 1, edgeR *P* value < 0.05]. Among these, 263 genes were upregulated, whereas 529 genes were downregulated in the GLA treated V27 sample, which was shown in the volcano plots (**Figure 1**). The biofilm-associated genes, whose functions involve adhesion, synthesis and release of EPS, as well as those whose functions are unclear but can affect biofilms, were the main targets we were searching for primarily in the RNA seq results.

The *fruA* (also known as *bepA*) gene putatively encodes a carbohydrate phosphotransferase system (PTS) permease (Protein ID: EFF35874.1) in the *E. faecium* E1162 strain involved in biofilm formation (Paganelli et al., 2016). Using the online software tblastn (https://blast.ncbi.nlm.nih.gov/Blast.cgi) for similarity analysis, the protein showed the highest similarity (93.01%) with the *EFUG_01291* gene in the reference genome (GenBank: GCA_000157615.1). RNA-seq showed a significant downregulation of the expression level of *EFUG_01291* gene (**Table 1**) in the V27 treated with GLA sample. In addition, the expression level of the *EFUG_01290* gene, which was 100% similar to FruB protein (Protein ID: EFF35875.1) in the *E. faecium* strain E1162, was also significant downregulation. The *fruB* gene has been reported to contribute to the fitness and virulence of *Streptococcus mutans* in human dental biofilms (Chakraborty et al., 2022). We have designated *EFUG_01291* and *EFUG_01290* genes as *fruA* and *fruB* genes, respectively.

**Table 1:** The biofilm-associated genes with statistical differences in the V27 treated with GLA relative to the V27 treated without GLA.

Gene ID	Product (*E. faecium* 1,231,408)	log2(Fold change)	Pfam annotation
EFUG_01291 (*fruA*)	Phosphotransferase system PTS protein	-2.25	PF02302: PTS system, lactose/cellobiose specific IIB subunit| PF02378: Phosphotransferase system, EIIC
EFUG_01290 (*fruB*)	PTS system protein	-1.56	PF00359: Phosphoenolpyruvate-dependent sugar phosphotransferase system, EIIA 2
EFUG_00477 (*sgrA*)	Cell wall surface adhesion protein	-1.16	No annotation
EFUG_00568 (*lpxtg-cwa*)	Cell wall surface adhesion protein	-1.84	PF00746:LPXTG cell wall anchor motif
EFUG_01915 (*tfpp*)	signal peptidase	-1.70	PF06750: Bacterial Peptidase A24 N-terminal domain
EFUG_01447 (*lafA*)	Glycosyl transferase	-1.59	PF13439: Glycosyltransferase family 4| PF00534: Glycosyl transferases group 1
EFUG_01446 (*lafB*)	Glycosyl transferase	-1.28	PF13439: Glycosyltransferase family 4| PF00534: Glycosyl transferases group 1
EFUG_01650 (*malP*)	Glycoside hydrolase	-1.45	PF03633: Glycosyl hydrolase family 65, C-terminal domain| PF03632: Glycosyl hydrolase family 65 central catalytic domain| PF03636: Glycosyl hydrolase family 65, N-terminal domain
EFUG_01694 (*fsrA*)	LytTR family transcriptional regulator	-1.62	PF04397: LytTR DNA-binding domain| PF00072: Response regulator receiver domain
EFUG_01696 (*fsrBD*)	Accessory gene regulator B	1.91	PF04647: Accessory gene regulator B
EFUG_00628 (*fsrC’*)	Histidine kinase	-1.49	PF14501: GHKL domain

The *sgrA* gene, encoding a nidogen-binding Leu-Pro-X-Thr-Gly, where X denotes any amino acid (LPXTG) surface adhesin protein (Protein ID: AFK59147.1) in the *E. faecium* DO strain implicated in biofilm formation (Hendrickx et al., 2007; Hendrickx et al., 2009a). This protein had the greatest similarity with the *EFUG_00477* gene (73.77%), whose expression level was downregulation in the V27 treated with GLA sample. We have designated *EFUG_00477* gene as *sgrA* gene.

Pfam annotation (http://pfam.xfam.org) showed that *EFUG_00568* gene encodes another LPXTG-motif protein. This gene was found to be significantly downregulated in the V27 treated with GLA sample, but it has not been previously reported to be associated with biofilm formation in *E. faecium*. Through sequence similarity analysis, this gene exhibited the highest similarity (96.33%) to the surface protein EF3314 (Protein ID: AAO82979.1) in *E. faecalis* V583, which has been linked to biofilm formation (Creti et al., 2009). We have designated *EFUG_00568* gene as *lpxtg-cwa* (LPXTG cell wall anchor) gene.

Pfam annotation showed that *EFUG_01915* gene encodes peptidase A24, which is a type 4 prepilin peptidase. It catalyzes the processing of type 4 prepilin to form type 4 pilus, which in turn are involved in a variety of functions, including toxin and enzyme secretion, gene transfer, and biofilm formation (LaPointe and Taylor, 2000). The expression of *EFUG_01915* gene was obviously down-regulated in the V27 treated with GLA sample in the study. We have designated *EFUG_01915* gene as *tfpp* (type 4 prepilin peptidase) gene.

Both *lafA* gene and *lafB* gene, encoding LafA (Protein ID: WP_002287605.1) and LafB (Protein ID: WP_002296953.1) glycosyltransferases in *E. faecium* DO strain, are involved in lipoteichoic acid (LTA) biosynthesis (Webb et al., 2009; Mello et al., 2020). These two proteins, LafA and LafB had the greatest similarity with the *EFUG_01447* gene (94.10%) and the *EFUG_01446* gene (99.71%), respectively. The closest homolog of *lafA* and *lafB* genes in the *E. faecalis* V583 strain is the biofilm-associated glycolipid synthesis B (*bgsB*) and *bgsA* genes, which are responsible for LTA anchor formation (Theilacker et al., 2011). While no studies have reported a relationship between *lafAB* genes and biofilm formation, the role of *bgsAB* genes in promoting biofilm formation has been well-established (Theilacker et al., 2011; Haller et al., 2014; Madsen et al., 2017). The expression of both *EFUG_01447* gene and *EFUG_01446* gene were obviously down-regulated in the V27 treated with GLA sample in the study. We have designated *EFUG_01447* and *EFUG_01446* genes as *lafA* and *lafB* genes, respectively.

The *malP* gene, encoding a maltose phosphorylase (Protein ID: WP_002413579.1) in *E. faecalis* OG1RF strain, is essential for the phosphorylation of maltose to α-D-glucose and glucose-1-phosphate (Sauvageot et al., 2017). This protein catalyzes the production of monosaccharides, which are essential for exopolysaccharide synthesis (Ali et al., 2022). It had the greatest similarity with the *EFUG_01650* gene (74.48%). The expression of the *EFUG_01650* gene was significantly down-regulated in the V27 treated with GLA sample in the study. We have designated the *EFUG_01650* gene as the *malP* gene.

### 3.3 GO and KEGG enrichment analysis

**Figure 2:**
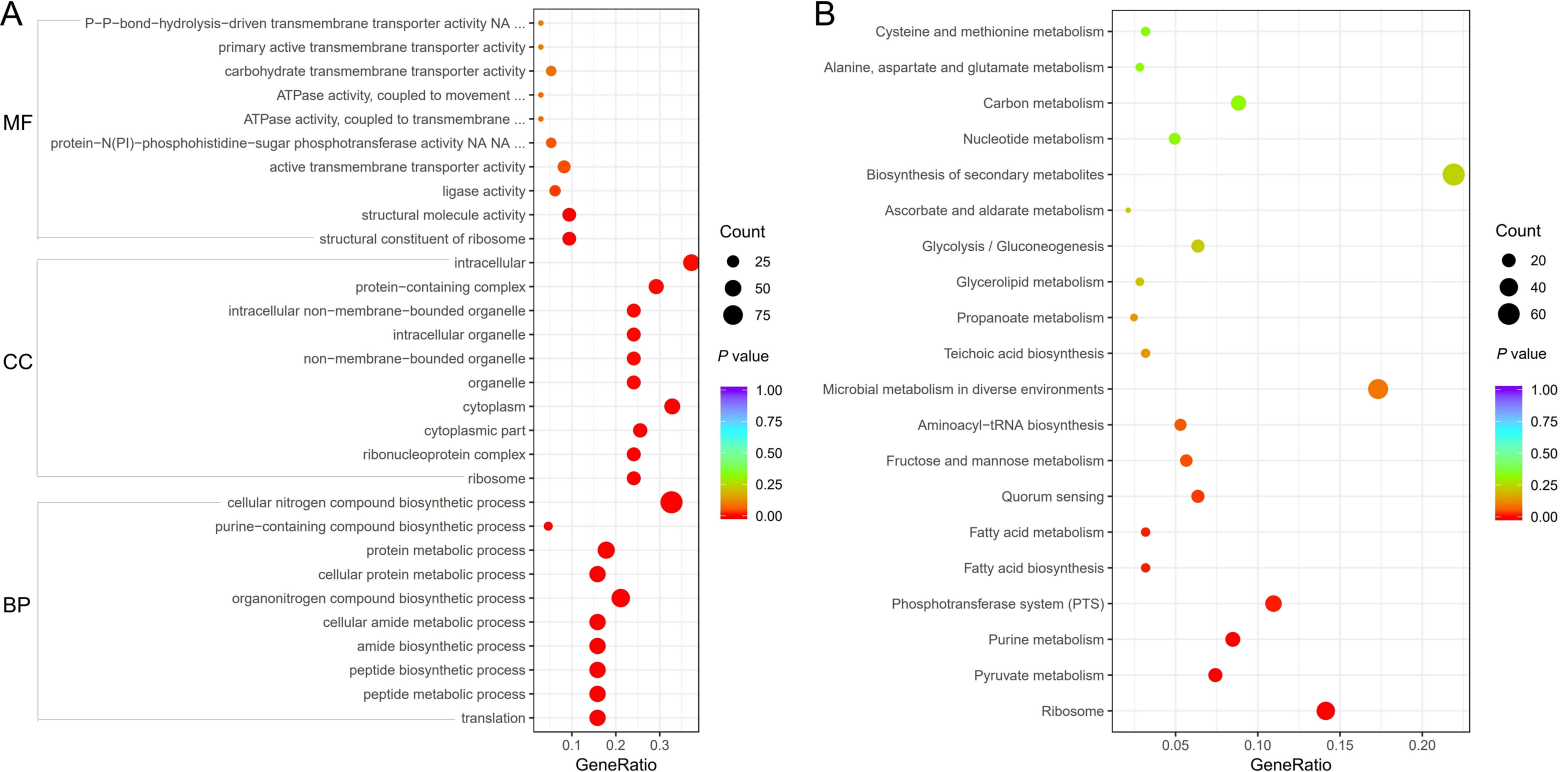
**(A)** Gene Ontology (GO) enrichment of differential expression genes between the V27 treated with GLA and V27 treated without GLA. MF, molecular function; CC, cell component; BP, biological process. **(B)** Kyoto Encyclopedia of Gene and Genomes (KEGG) pathway enrichment of differential expression genes between the V27 treated with GLA and V27 treated without GLA.

Three categories include cell component, biological process, and molecular function in GO enrichment analysis. The Gene Ratio, the count, and the corresponding *P* values for each GO ID comparing the V27 treated with GLA to the V27 treated without GLA samples, were presented in **Figure 2A**. The percentage of upregulated genes in GO enrichment analysis was shown in **Supplementary Table S3**. Among the top ten enriched cell components, including ribosome, ribonucleoprotein complex, cytoplasmic part, cytoplasm, organelle, non-membrane-bounded organelle, intracellular organelle, intracellular non-membrane-bounded organelle, protein-containing complex, and intracellular, at least 86.7% of genes were upregulated. Similarly, six biological processes exhibited a greater than 90% upregulation of genes, including translation, peptide metabolic process, peptide biosynthetic process, amide biosynthetic process, cellular amide metabolic process, and cellular protein metabolic process. Two additional processes showed upregulation of genes between 80-90%, and two processes showed upregulation of genes between 60-70%. Of the four molecular function categories displaying statistically significant differences, structural constituent of ribosome and structural molecule activity showed an upregulation of 93.8% of genes, while ligase activity had an upregulation of genes of 66.7%. However, active transmembrane transporter activity showed a significantly reduced upregulation of genes of only 39.3%.

Enrichment analysis of KEGG pathways identified eight pathways with statistically significant enrichment (*P* < 0.05), fewer than those observed in GO analysis. **Figure 2B** showed the detailed results for these enriched pathways, including the Gene Ratio, the count, and the corresponding *P* values (obtained by comparing V27 samples treated with GLA to untreated V27 samples). Among these enriched pathways, only the ribosome pathway exhibited a significantly higher proportion of upregulated genes (92.5%). The remaining pathways showed lower proportions of upregulated genes, all below 50%. Specifically, pathways such as pyruvate metabolism, purine metabolism, the PTS, and fructose and mannose metabolism displayed upregulated gene proportions between 25% and 43%, whereas fatty acid biosynthesis and fatty acid metabolism showed no upregulated genes. Detailed upregulated proportions can be found in **Supplementary Table S4**.

**Figure 3:**
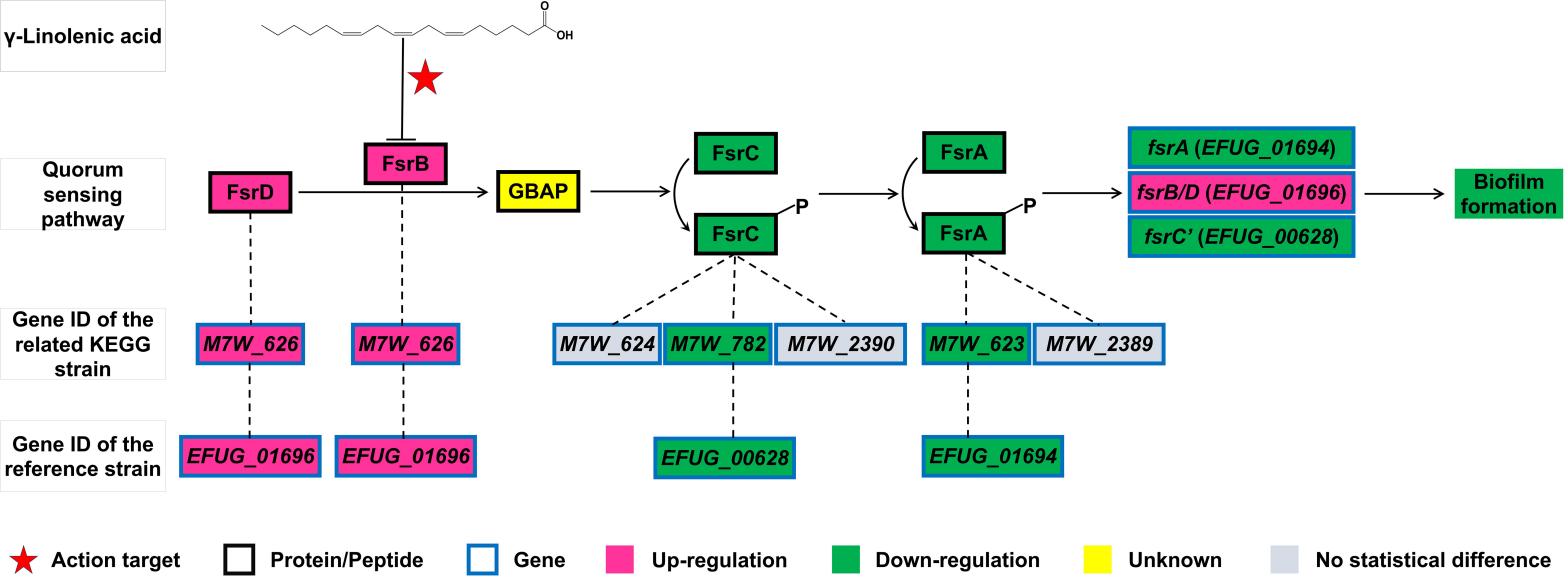
The mechanism of action of GLA on the Fsr quorum sensing pathway and its associated genes in this study.

The PTS and fructose and mannose metabolism pathways both contain the genes *fruA* and *fruB*. The Fsr quorum sensing system is involved in biofilm formation. In the KEGG strain *E. faecium* NRRL B-2354 (GenBank: CP004063.1), the regulatory genes for FsrA protein were *M7W_623* and *M7W_2389*, for FsrB/D protein/peptide was *M7W_626* gene, and for FsrC protein were *M7W_624*, *M7W_782*, and *M7W_2390* genes (**Figure 3**). In this study, the reference strain *E. faecium* 1,231,408 exhibited downregulation of the *EFUG_01694* and *EFUG_00628* genes (**Table 1**), which corresponded to *M7W_623* and *M7W_782* genes in the KEGG strain *E. faecium* NRRL B-2354, respectively. Conversely, the *EFUG_01696* gene, corresponding to the *M7W_626* gene in the KEGG strain *E. faecium* NRRL B-2354, showed upregulation. We have designated *EFUG_01694* and *EFUG_01696* genes as *fsrA* and *fsrB* genes, respectively. Based on existing nomenclature, the *M7W_624* gene, which corresponds to the *EFUG_01695* gene in the reference genome, is annotated as *fsrC*. Therefore, we have designated *M7W_782*, which corresponds to *EFUG_00628* in the reference genome, as *fsrC’*.

### 3.4 Validation by biofilm-associated genes of VRE-fm isolates by qRT-PCR

**Figure 4:**
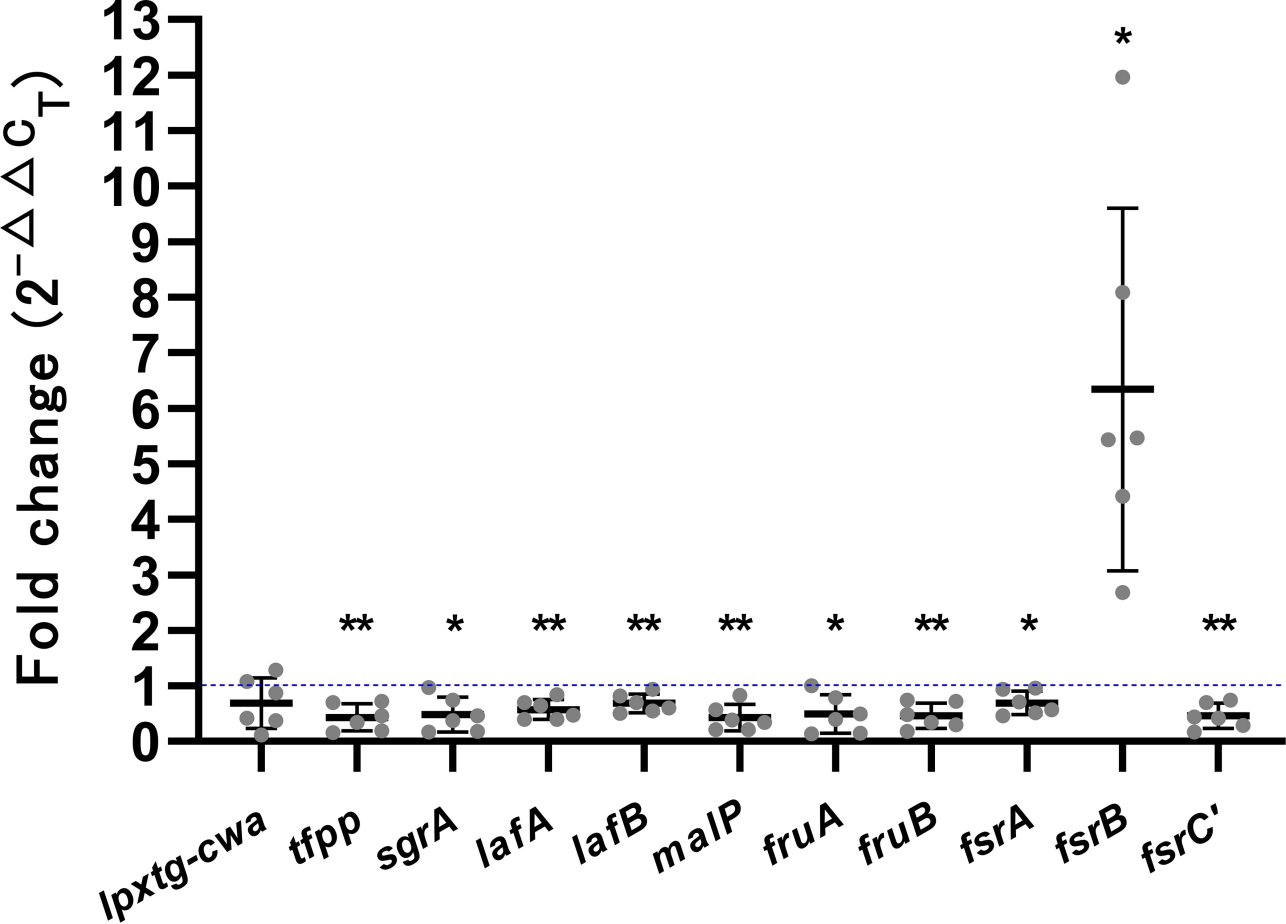
The expression levels of biofilm-related genes in mature biofilms of the VRE-fm isolates treated with γ-linolenic acid (GLA) comparing to the control group. *P < 0.05, and **P < 0.01.

To validate transcriptome sequencing results, genes involved in biofilm expression of six VRE-fm isolates (V05, V06, V08, V09, V22, and V27) were selected as targets for qRT-PCR analysis, as shown in **Figure 4**. Compared to the control group, GLA down-regulated the gene expression of *lpxtg-cwa* (0.69-fold), *tfpp* (0.43-fold), *sgrA* (0.48-fold), *lafA* (0.57-fold), *lafB* (0.69-fold), *malP* (0.43-fold), *fruA* (0.49-fold), *fruB* (0.46-fold), *fsrA* (0.69-fold), and *fsrC’* (0.46-fold), while up-regulated the gene expression of *fsrB* (6.34-fold). The qRT-PCR results showed a high concordance with the transcriptomic data, with all gene expression changes exhibiting statistical significance except for the decrease in *lpxtg-cwa*, which was not statistically different.

**Figure 5:**
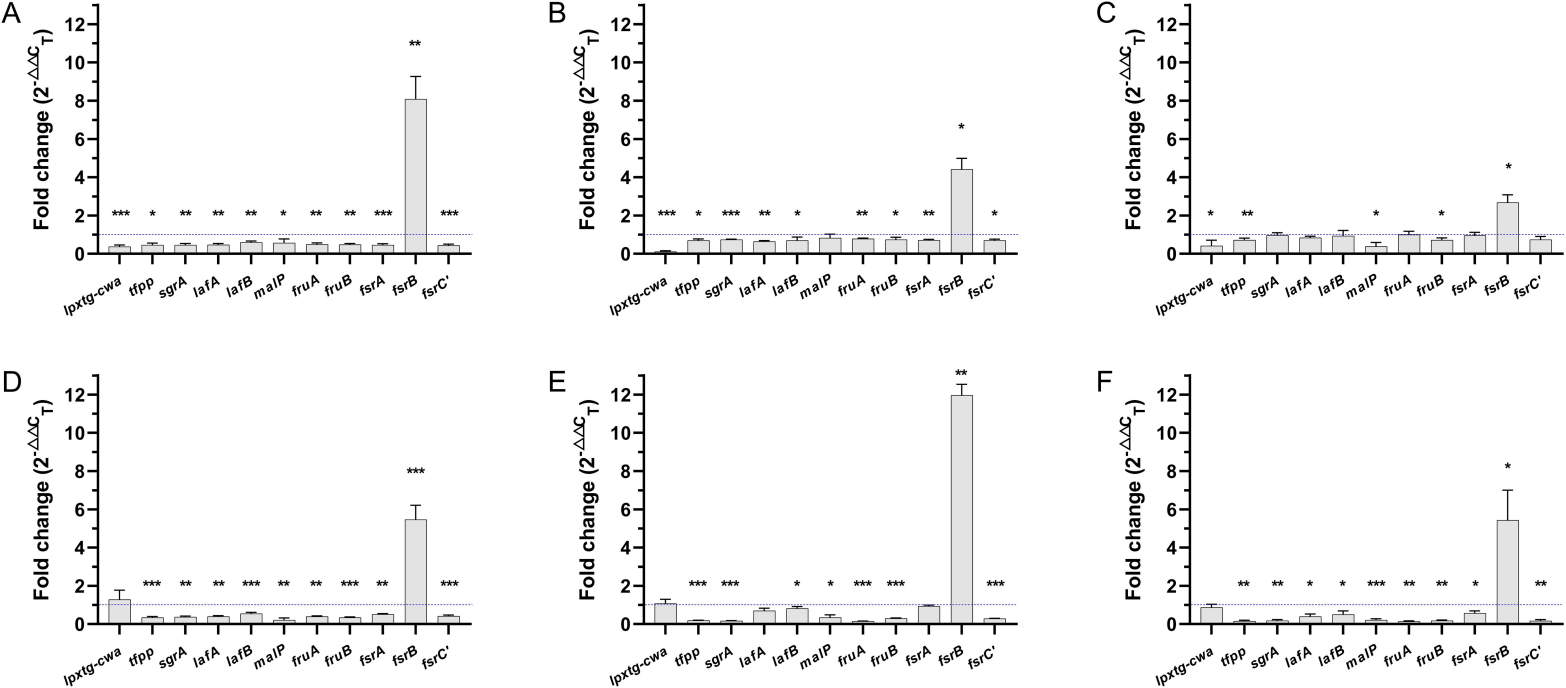
The expression levels of biofilm-related genes in mature biofilms of each VRE-fm isolate treated with γ-linolenic acid (GLA) comparing to the control group **(A)** V05; **(B)** V06; **(C)** V08; **(D)** V09; **(E)** V22; **(F)** V27. *P < 0.05, **P < 0.01, and ***P < 0.001.

**Figure 5** further showed the regulatory effects of GLA on the expression levels of biofilm-related genes in different VRE-fm isolates. Genes including *tfpp*, *sgrA*, *lafA*, *lafB*, *malP*, *fruA*, *fruB*, *fsrA*, and *fsrC* were downregulated to varying degrees in all isolates. Conversely, *fsrB* gene was upregulated to varying degrees in all isolates. The *lpxtg-cwa* gene exhibited inconsistent expression patterns across the six isolates; downregulation was observed in V05, V06, V08, and V27, whereas upregulation was observed in V09 and V22.

### 3.5 GLA affects biofilm-related genes in *Enterococcus faecalis*

**Figure 6:**
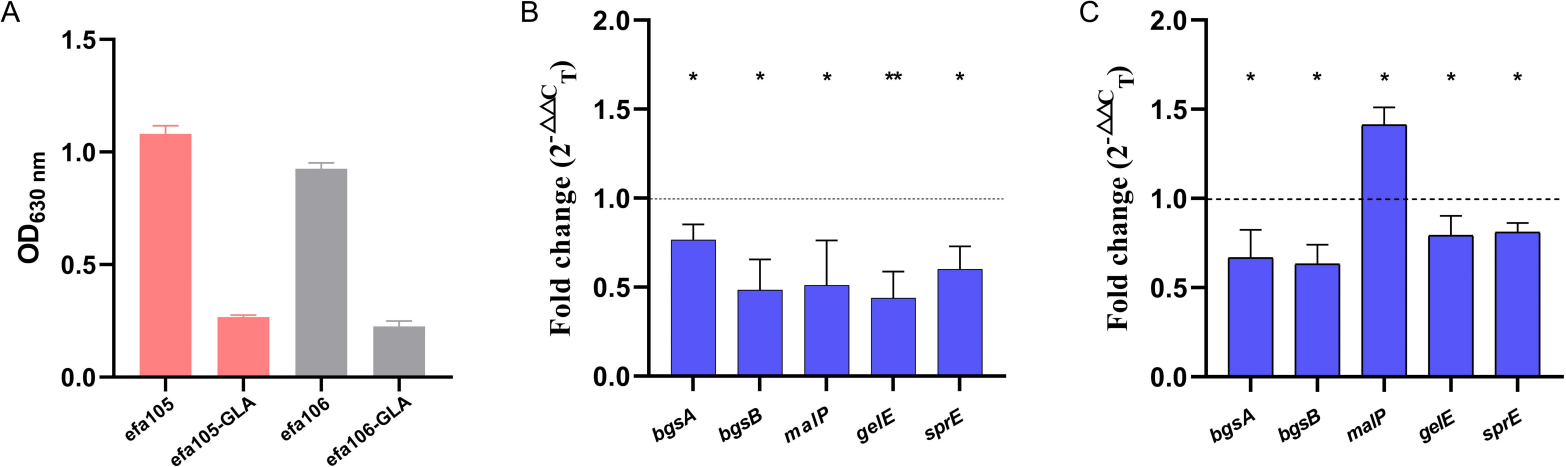
**(A)** The biofilm biomass of two clinical isolates of *E*. *faecalis* (efa105 and efa106) before and after treated with γ-linolenic acid (GLA); The expression levels of biofilm-related genes in mature biofilms of **(B)** efa105 and **(C)** efa106 treated with γ-linolenic acid (GLA) comparing to the control group. *P < 0.05, and **P < 0.01.

Because the effects of certain genes on biofilm formation in *E. faecium* were predicted from homologous genes in *E. faecalis*, we validated these predictions by investigating the effect of GLA on these homologous genes in *E. faecalis*. **Figure 6A** showed that two clinical isolates of *E. faecalis* (efa105 and efa106) both exhibited strong biofilm formation ability. After treatment with GLA, the biofilm biomass of both isolates decreased by 75.2% and 75.6%, respectively. **Figures 6B, C** showed the effect of GLA on the gene expression levels of *bgsA*, *bgsB*, efa-*malP*, *gelE*, and *sprE* in isolates efa105 and efa106, with the latter two genes being positively regulated by the Fsr quorum sensing system. All gene expression levels in isolate efa105 were downregulated, while in isolate efa106, all gene expression levels except for efa-*malP* were also downregulated.

## 4 Discussion

Biofilm formation significantly contributes to the pathogenesis of *Enterococcus*, especially in infective endocarditis and urinary tract infections, where it promotes bacterial persistence and resistance to antibiotics (Wei et al., 2024). An intriguing study revealed that a unique metabolite, putatively a short-chain fatty acid (SCFA) produced by Bacteroides spp. (dominant members of the gut microbiota), demonstrated a strong association with VRE-fm decolonization (Deleu et al., 2021; MaChado et al., 2021). Similarly, our previous study demonstrated that essential fatty acids can inhibit and eradicate *E. faecium* biofilms. Preliminary mechanistic investigations revealed a significant downregulation of *atlA* gene expression (Wei et al., 2023). However, the downregulation of a single gene might not be sufficient to explain the substantial eradication of preformed biofilms. Therefore, in this study, we employed transcriptome analysis to further elucidate the mechanisms.

Transcriptome analysis revealed a significant downregulation in the expression levels of genes associated with biofilm formation, including *fruA*, *fruB*, *sgrA*, *lpxtg-cwa*, *tfpp*, *lafA*, *lafB*, *malP*, *fsrA*, and *fsrC’*, while a significant upregulation was observed in the expression of *fsrBD*. (**Table 1**). Validation by qRT-PCR in six VRE-fm isolates confirmed the significant changes in the expression levels of all genes except for *lpxtg-cwa*, with statistical significance (**Figure 4**).

Fernanda et al. reported that deletion of the *fruA* (*bepA*) gene in *E. faecium* strain E1162 impaired biofilm formation and metabolism of β-methyl-D-glucoside. Since β-glucoside metabolism is linked to the metabolism of glycosaminoglycans, which are exposed on injured heart valves where bacteria attach and form vegetations, the authors propose that the PTS permease FruA (BepA) influences biofilm formation through this pathway (Paganelli et al., 2016). The *fruAB* genes constitute an operon, and the *fruB* gene is coordinately regulated with the *fruA* gene (Burne et al., 1999; Chakraborty et al., 2022). In *S. mutans*, the *fruB* gene encodes an endolevanase FruB, that enhances levan metabolism, and provides the bacteria with energy and carbon sources. It plays a crucial role in biofilm formation and pathogenesis (Chakraborty et al., 2022). The current study found that GLA simultaneously downregulated the expression of both *fruA* and *fruB* genes. However, the involvement of *fruB* in biofilm formation in *E. faecium* remains unclear and requires further investigation. Additionally, it is yet to be confirmed whether GLA downregulates *fruAB* expression by inhibiting their promoter.

Biofilm formation in *Enterococcus* species is initiated by adhesion, a critical step that sets the stage for subsequent biofilm development (Ch'ng et al., 2019). SgrA belongs to the LPxTG-type surface proteins, a class of proteins characterized by a C-terminal LPxTG-like motif (Hendrickx et al., 2009a). This motif is recognized and cleaved by a transpeptidase (sortase), which covalently anchors the surface protein to the cell wall peptidoglycan (Hendrickx et al., 2009b). SgrA has been shown to bind to the extracellular matrix molecules nidogens and fibrinogen. Deletion of the *sgrA* gene significantly impairs biofilm formation. This study found that GLA significantly decreased the expression of *sgrA* gene (Hendrickx et al., 2009a), suggesting a potential mechanism for the antibiofilm activity of GLA. Although a decrease in the expression of another LPxTG-type surface protein-encoding gene (*lpxtg-cwa*) was observed in some individual isolates, this reduction was not statistically significant for all VRE-fm isolates.

Another important structure involved in *Enterococcus* adhesion is pilus (Hendrickx et al., 2009b). Type IV pilus formation relies on the cleavage of type IV prepilins by type IV prepilin peptidase (TFPP). Following cleavage, type IV prepilins not only contribute to type IV pilus assembly but also participate in a variety of biological processes, including toxin and enzyme secretion, gene transfer, and biofilm formation (LaPointe and Taylor, 2000). In this study, GLA significantly downregulated the expression of the *tfpp* gene. Therefore, GLA can also inhibit biofilm formation by interfering with type IV pilus formation.

Both *lafA* and *lafB* genes, encoding glycosyltransferase, are involved in the biosynthesis of lipoteichoic acid (LTA) (Mello et al., 2020). The glycolipid anchor of LTA is able to insert into eukaryotic membranes. In a study of *E. faecalis*, deletion of the *bgsB* and *bgsA* genes, which are homologous to *lafA* and *lafB* genes, significantly impaired biofilm formation in a rat model of infective endocarditis (Haller et al., 2014). In this study, GLA significantly downregulated the expression of *lafA* and *lafB* genes. Based on this homology, we further investigated two clinical isolates of *E. faecalis* and found that GLA could also downregulate the expression of *bgsA* and *bgsB* genes. However, the potential role of *lafA* and *lafB* genes in influencing biofilm formation in *E. faecium* remains to be elucidated and warrants further investigation. Suelen et al. reported that deletion of the *lafB* gene resulted in increased susceptibility to daptomycin (Mello et al., 2020). Therefore, GLA may enhance daptomycin susceptibility in *E. faecium*.

EPS production plays a crucial role in biofilm maturation. Our previous study has demonstrated that GLA inhibits eDNA release by downregulating the expression of *atlA* gene (Wei et al., 2023). In this study, we further observed that GLA downregulates the expression of the *malP* gene. In *E. faecalis*, the homologous gene efa-*malP* is essential for the phosphorylation of intracellular maltose to α-D-glucose and glucose-1-phosphate. Downregulation of *malP* results in a decrease in the availability of monosaccharides needed for exopolysaccharide synthesis (Ali et al., 2022). Based on this homology, we also investigated the expression of the efa-*malP* gene in *E. faecalis*. We observed contrasting effects of GLA on efa-*malP* expression in two *E. faecalis* isolates, with a decrease in one isolate and an increase in the other. Therefore, further studies with a larger sample size are needed to confirm the effect of GLA on efa-*malP* gene expression in *E. faecalis*.

Quorum sensing is a type of cell-to-cell communication used by bacteria. It allows bacteria to coordinate their behavior based on the density of their population (Mukherjee and Bassler, 2019). The Fsr quorum sensing system plays a role in biofilm formation in *Enterococcus* (Tsikrikonis et al., 2012; Ali et al., 2017). The FsrD propeptide is processed by FsrB to produce gelatinase biosynthesis-activating pheromone (GBAP) (**Figure 3**). Accumulated GBAP is sensed by the sensor protein, FsrC, leading to its phosphorylation and activation of the response regulator, FsrA. Activated FsrA promotes the expression of the *fsr* locus, *gelE*, and *sprE* genes. Upregulation of *gelE* expression, which encodes a gelatinase, promotes biofilm formation in *E. faecalis (* Ali et al., 2017 *)*. The precise mechanisms by which the Fsr system promotes biofilm formation in *E. faecium* remain unclear. However, a study has shown that deletion of the *fsrB* gene inhibits biofilm formation (Tsikrikonis et al., 2012). In this study, we demonstrated that GLA decreased the expression of the *fsrA* and *fsrC’* genes but significantly increased the expression of the *fsrB* gene. This result contradicts the expectation that reduced *fsrA* expression would inhibit the expression of the *fsr* locus. A previous study has suggested that the function of FsrB can be inhibited by ambuic acid, thereby reducing the production of the signaling molecule GBAP (Nakayama et al., 2009). Therefore, we hypothesize that GLA, similar to Ambuic Acid, can also inhibit the function of FsrB, thereby suppressing biofilm formation. The observed increase in *fsrB* gene expression may be attributed to negative feedback regulation resulting from FsrB inhibition. Further investigation is required to elucidate the specific mechanisms involved in this interaction. While the specific genes regulated by the Fsr system in *E. faecium* remain unclear, this system is known to regulate the expression of *gelE* and *sprE* genes in *E. faecalis*. Therefore, we investigated the effects of GLA on the expression of *gelE* and *sprE* in *E. faecalis*. The results demonstrated that GLA reduced the expression of *gelE* and *sprE* genes in two *E. faecalis* clinical isolates. These findings provide further evidence that GLA inhibits the Fsr system, highlighting its potential as a novel anti-biofilm agent.

In addition to downregulating biofilm-related genes, GLA also alters metabolic pathways, further supporting its biofilm eradicating effects. GO analysis revealed a significantly higher proportion of upregulated genes across all GO IDs except for the active transmembrane transporter activity, suggesting a more active metabolic state in GLA-treated cells compared to biofilm-embedded cells. This finding aligns with our understanding that cells within biofilms exhibit a slower metabolic rate (Vestby et al., 2020). Furthermore, KEGG analysis identified specific metabolic pathways that were significantly downregulated by GLA, including purine, phosphotransferase system, and fatty acid metabolism. These molecules may play a crucial role in cell survival, repair, and biofilm formation (Zhang et al., 2024).

In summary, we investigated the effects of GLA on VRE-fm using transcriptomic analysis and qRT-PCR. Our results revealed that GLA significantly reduced the expression of numerous biofilm-associated genes in VRE-fm. Furthermore, GLA inhibited the Fsr quorum sensing system by suppressing the function of FsrB. The expression of *bgsB* and *bgsA* genes, which are the homologs of *lafA* and *lafB* genes, along with the Fsr-regulated genes *gelE* and *sprE* in *E. faecalis*, were also found to be downregulated by GLA. GO analysis revealed a more active metabolic state in GLA-treated VRE-fm cells compared to biofilm-embedded cells, suggesting a shift in metabolic activity. KEGG analysis identified specific metabolic pathways were significantly downregulated by GLA. Overall, these findings demonstrate that GLA effectively targets multiple aspects of biofilm formation in VRE-fm, including the downregulation of key biofilm-related genes, the inhibition of quorum sensing systems, and the modulation of metabolic pathways. GLA emerges as a promising candidate for eradicating *Enterococcus* biofilms.

## Data Availability

The original contributions presented in the study are publicly available. This data can be found here: https://www.ncbi.nlm.nih.gov/sra/PRJNA1178430.
