# Supplementary material for: Transcriptome analysis reveals the molecular mechanism of γ-linolenic acid eradicating the biofilm of vancomycin-resistant Enterococcus faecium
Source: Front Cell Infect Microbiol. 2025 Jan 31;15:1525581. doi: 10.3389/fcimb.2025.1525581 (PMC11826239; doi:10.3389/fcimb.2025.1525581)
Supplement: Supplementary file 1 [file Table1.docx]

Supplementary Material

# Supplementary Figures and Tables

## Supplementary Figures


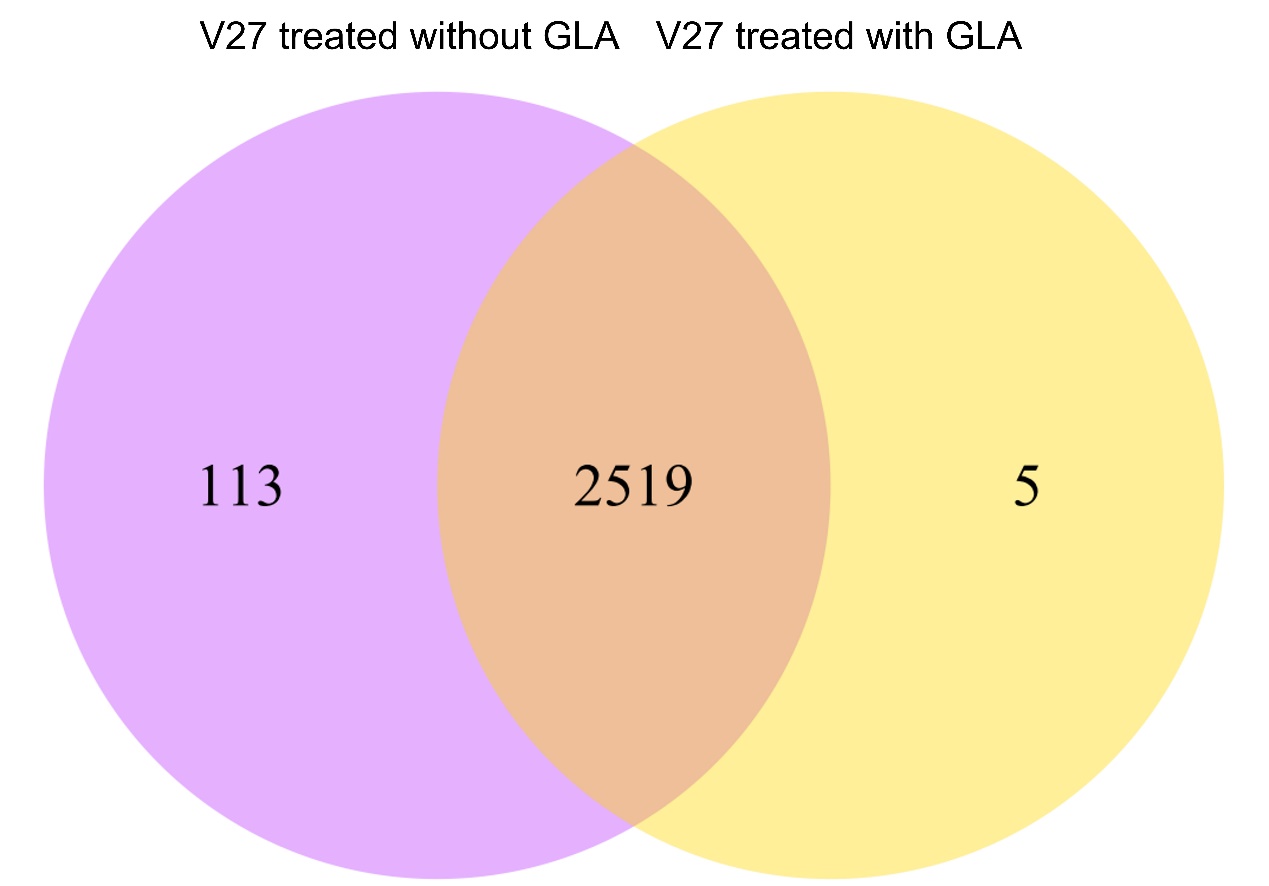


**Supplementary Figure 1.** The Venn diagram of V27 treated with GLA and V27 treated without GLA samples.

## Supplementary Tables

**Table S1** List of primers used in this study for *E. faecium*

| **Primers** | **Primer sequence (5′–3′)** | **Product length (bp)** | **References** |
| --- | --- | --- | --- |
| *gdhA*-F  *gdhA*-R | TGAAGCAGGCGTGTGGTATT  GGCGACACGCATGGTAAATC | 164 | This study (NZ_CP118955.1) |
| *lpxtg-cwa*-F  *lpxtg-cwa*-R | ATCCTGCGACAGATCCTCCT  GCCAAGTCTTTCGCGTTCTC | 187 | This study (GG688625.1) |
| *tfpp*-F  *tfpp*-R | TGTCAAATGCTGTCGTTCGC  CCAGGCAAAAAGACCACCAC | 148 | This study (GG688625.1) |
| *sgrA*-F  *sgrA*-R | AGACGTACAATGGACCGCTG  TGCTATCCGTACTGCTTGGC | 185 | This study (GG688625.1) |
| *lafA*-F  *lafA*-R | ACACCTTGTGTCGTGGAAGG  TGCTGGTGCAAAGTCTTCGT | 99 | This study (GG688625.1) |
| *lafB*-F  *lafB*-R | GCGGACTATCTGATCACGCC  AATCGGCACACGGATACCAT | 72 | This study (GG688625.1) |
| *malP*-F  *malP*-R | ACGGAAAAGCAGTCGTTCCT  ACTGTCCATGTCTCGCCTTG | 94 | This study (GG688625.1) |
| *fruA*-F  *fruA*-R | CCGATTGCTGCTTGTATGGC  TCGGGTCAGATACCGCAAAC | 175 | This study (GG688625.1) |
| *fruB*-F  *fruB*-R | CTCAGCACACTCTCCAGACG  GCTGGCGTAGTGGCTTCTTT | 71 | This study (GG688625.1) |
| *fsrA*-F  *fsrA*-R | AACTTCTCAATGCGGCCAAG  ACGCCATCCATTTCACTGTC | 88 | This study (GG688625.1) |
| *fsrB*-F  *fsrB*-R | GGCGGAAACAGGAATCACCT  GTGTCCACAGCAGACAAACG | 98 | This study (GG688625.1) |
| *fsrC’*-F  *fsrC’*-R | CGTGTAGCCGATCAGAGTCC  CGTCGGATTTGCCTTGTTCG | 90 | This study (GG688625.1) |

**Table S2** List of primers used in this study for *E. faecalis*

| **Primers** | **Primer sequence (5′–3′)** | **Product length (bp)** | **References** |
| --- | --- | --- | --- |
| efa-*gdhA*-F  efa-*gdhA*-R | TAGGCGCTCGTGAAATTGGT  CTTGCTTGGCTTCCCCAGTA | 109 | Submitted for publication (NC_017316.1) |
| *bgsA*-F  *bgsA*-R | TTGCGCGACAGTTACCAGAG  GCATAGGCCCCTTCGATGAC | 157 | This study (NC_017316.1) |
| *bgsB*-F  *bgsB*-R | ATTTTGTCAGCGCCTCAACG  TCTGCAACACATTGAACGCC | 82 | This study (NC_017316.1) |
| efa-*malP*-F  efa-*malP*-R | TGAAGCAAAAGCTGGGCAAG  TGTTGGGCTTCGTCATCTCC | 87 | This study (NC_004668.1) |
| *gelE*-F  *gelE*-R | TGGGATGGAAAAGCAATGCG  AAACCGGCAGTATGTTCCGT | 122 | [1] |
| *sprE*-F  *sprE*-R | ATCGTTCCTGCCGAAAGTCA  AAGGCGCTTCGGTTGTATCT | 94 | This study (NC_017316.1) |

**Table S3** The percentage of upregulated genes in GO enrichment analysis

| **Category** | **GO ID** | **Description** | **Percentage of upregulated genes (%)** |
| --- | --- | --- | --- |
| CC | GO:0005840 | Ribosome | 93.9 |
| CC | GO:1990904 | Ribonucleoprotein complex | 93.9 |
| CC | GO:0044444 | Cytoplasmic part | 88.6 |
| CC | GO:0005737 | Cytoplasm | 86.7 |
| CC | GO:0043226 | Organelle | 93.9 |
| CC | GO:0043228 | Non-membrane-bounded organelle | 93.9 |
| CC | GO:0043229 | Intracellular organelle | 93.9 |
| CC | GO:0043232 | Intracellular non-membrane-bounded organelle | 93.9 |
| CC | GO:0032991 | Protein-containing complex | 87.5 |
| CC | GO:0005622 | Intracellular | 88.2 |
| BP | GO:0006412 | Translation | 93.8 |
| BP | GO:0006518 | Peptide metabolic process | 93.8 |
| BP | GO:0043043 | Peptide biosynthetic process | 93.8 |
| BP | GO:0043604 | Amide biosynthetic process | 93.8 |
| BP | GO:0043603 | Cellular amide metabolic process | 93.8 |
| BP | GO:1901566 | Organonitrogen compound biosynthetic process | 84.4 |
| BP | GO:0044267 | Cellular protein metabolic process | 93.8 |
| BP | GO:0019538 | Protein metabolic process | 88.9 |
| BP | GO:0072522 | Purine-containing compound biosynthetic process | 64.3 |
| BP | GO:0044271 | Cellular nitrogen compound biosynthetic process | 66.7 |
| MF | GO:0003735 | Structural constituent of ribosome | 93.8 |
| MF | GO:0005198 | Structural molecule activity | 93.8 |
| MF | GO:0016874 | Ligase activity | 66.7 |
| MF | GO:0022804 | Active transmembrane transporter activity | 39.3 |

**Table S4** The percentage of upregulated genes in KEGG enrichment analysis

| **KEGG ID** | **Description** | **Percentage of upregulated genes (%)** |
| --- | --- | --- |
| efm03010 | Ribosome | 92.5 |
| efm00620 | Pyruvate metabolism | 42.9 |
| efm00230 | Purine metabolism | 25.0 |
| efm02060 | Phosphotransferase system (PTS) | 25.8 |
| efm00061 | Fatty acid biosynthesis | 0 |
| efm01212 | Fatty acid metabolism | 0 |
| efm02024 | Quorum sensing | 27.8 |
| efm00051 | Fructose and mannose metabolism | 37.5 |

**References**

1. Wei, M., Wang, P., Li, T., Wang, Q., Su, M., Gu, L., Wang, S., 2023. Antimicrobial and antibiofilm effects of essential fatty acids against clinically isolated vancomycin-resistant *Enterococcus faecium*. Front. Cell. Infect. Microbiol. 13, 1266674. https://doi.org/10.3389/fcimb.2023.1266674.
